# The efficacy and safety of Fufangdanshen tablets (*Radix Salviae miltiorrhizae* formula tablets) for mild to moderate vascular dementia: a study protocol for a randomized controlled trial

**DOI:** 10.1186/s13063-016-1410-5

**Published:** 2016-06-08

**Authors:** Jinzhou Tian, Jing Shi, Mingqing Wei, Renan Qin, Jingnian Ni, Xuekai Zhang, Ting Li, Yongyan Wang

**Affiliations:** BUCM Neurology Centre, Dongzhimen Hospital of Beijing University of Chinese Medicine, Beijing, China; Hutchison Whampoa Guangzhou Baiyunshan Chinese Medicine Company Limited, Guangzhou, China; Institute of Clinical Medicine, China Academy of Chinese Medical Sciences, Beijing, China

**Keywords:** Vascular dementia, Randomized controlled trial, Fufangdanshen tablets, Chinese medicine

## Abstract

**Background:**

Vascular dementia (VaD) is the second most common subtype of dementia after Alzheimer's disease (AD). Currently, there are no medications approved for treating patients with VaD. Fufangdanshen (FFDS) tablets (*Radix Salviae miltiorrhizae* formula tablets) are a traditional Chinese medicine that has been reported to improve memory. However, the existing evidence for FFDS tablets in clinical practice derives from methodologically flawed studies. To further investigate the safety, tolerability, and efficacy of FFDS tables in the treatment of mild to moderate VaD, we designed and reported the methodology for a 24-week randomized, double-blind, parallel, multicenter study.

**Methods/design:**

This ongoing study is a double-blind, randomized, parallel placebo-controlled trial. A total of 240 patients with mild to moderate VaD will be enrolled. After a 2-week run-in period, the eligible patients will be randomized to receive either three FFDS or placebo tablets three times per day for 24 weeks, with a follow-up 12 weeks after the last treatment. The primary efficacy measurement will be the Alzheimer’s Disease Assessment Scale-cognitive subscale (ADAS-cog) and the Clinician Interview-Based Impression of Change (CIBIC-plus). The secondary efficacy measurements will include the Mini Mental State Examination (MMSE) and activities of daily living (ADL). Adverse events will also be reported.

**Discussion:**

This randomized trial will be the first rigorous study on the efficacy and safety of FFDS tablets for treating cognitive symptoms in patients with VaD using a rational design.

**Trial registration:**

ClinicalTrials.gov: NCT01761227. Registered on 2 January 2013.

## Background

Vascular dementia (VaD) is the second most common subtype of dementia after Alzheimer's disease (AD) [1], and it accounts for 11.1–15.8 % of all cases of dementia worldwide [2, 3]. The EURODEM Prevalence Research Group compared the prevalence of VaD in five datasets from Europe (Finland, Italy, Sweden, and two from the United Kingdom) and found that the prevalence ranged from 0.0–1.6 % for those aged between 60 and 70 years and increased to 2.8–9.2 % for subjects aged 80–90 years. The annual incidence rate was estimated to be 3.79 per 1000 among non-demented populations. At present, the treatment of VaD focuses on primary and secondary prevention strategies because randomized clinical trials in VaD have not been able to demonstrate clinically relevant symptomatic improvement. Additionally, it has not yet been possible to establish disease-modifying effects in VaD syndrome [4]. Thus, the development of an effective treatment for VaD is important.

Fufangdanshen tablets (FFDS), a traditional herbal medicine approved by the China Food and Drug Administration (CFDA) in 2008 (Number: [2008]1919), are extracted from the Chinese herbs *Salvia miltiorrhiza*, *Panax notoginseng*, and *Borneolum syntheticum* and contain tanshinone, salvianolic acid, panax notoginsenosides, ginsenoside Rb1, ginsenoside Rg1, and borneol. The tablets are used to treat patients with VaD. Preclinical studies have shown that tanshinone can improve the impaired learning and memory induced by Aβ1-40 in rat models of AD [5], inhibiting AD-induced expression levels of inducible nitric oxide synthase (iNOS) and matrix metalloproteinase II (MMP-2), reducing toxic free radicals, and suppressing oxidative injury in AD rats [6]. Salvianolic acid can inhibit glutamate release and anti-cerebral ischemic effects [7]. Borneol can improve the permeability of the blood-brain barrier [8].

Many studies have reported that FFDS tablets can improve the memory of demented mice [9] and improve the impairment of spatial discrimination and memory impairment in rat models of AD. The mechanisms may involve improvement in the brain choline acetyl transferase (ChAT) activity that is decreased in AD rats and induced by Aβ [10], lowering the toxicity of excitatory amino acids [11] and increasing the expression of vascular endothelial growth factor (VEGF) in the brains of rats during chronic cerebral ischemia [11]. Additionally, FFDS tablets could improve the learning and memory capabilities in rat models of VaD, increase the activity of superoxide dismutase, and reduce neuron apoptosis in the hippocampus [12].

A phase II clinical trial on the efficacy of FFDS tablets in VaD was carried out in five centers. The trial enrolled 231 patients; all patients were randomized to the FFDS tablets group (115 patients) or the dihydroergotoxine mesylate tablets group (116 patients). Scores on the Mini Mental State Examination (MMSE) [13] and activities of daily living significantly improved in both groups compared with baseline (*P* < 0.001). However, there were some limitations to this study. First, there was no placebo group. As all subjects knew that they were being treated with one of two drugs and all of the individuals who assessed the patients knew this as well, the results may have been influenced by a positive response bias. Second, the sample size was relatively small. Third, this previous study only followed up patients for 12 weeks, and this period of follow-up was too short to detect the effects of the drugs.

To further investigate the safety, tolerability, and efficacy of FFDS tablets in the treatment of mild to moderate VaD, we designed and report the methodology for a 24-week randomized, double-blind, parallel, multicenter clinical study.

## Methods/design

### Study design

This ongoing study was designed as a randomized, double-blind, parallel, placebo-controlled, multicenter trial (six centers). It involves a single-blind run-in (and washout) period using placebo only (2 weeks) and a double-blind treatment phase after randomization (24 weeks). The study is being carried out at six centers in China. It has been approved by the China Food and Drug Administration (code: SFDA [2008] I919) and also by the Institutional Review Board of Dongzhimen Hospital, Beijing University of Chinese Medicine and has been registered with ClinicalTrials.gov (ClinicalTrials.gov: NCT01761227). If there is any amendment to the protocol, approval must again be sought from the Ethics Committee. The patients and responsible caregivers will provide written informed consent. The study will be conducted according to Good Clinical Practice Guidelines and the principles of the Declaration of Helsinki. The protocol design is based on the guidelines of Consolidated Standards of Reporting Trials (CONSORT), and the study results will also be reported according to these guidelines.

### Participants

This trial will enroll both outpatient and inpatient Chinese-speaking males and females aged 45–80 years old, weighing 45–90 kilograms, and meeting the diagnostic criteria of probable VaD established according to the Diagnostic and Statistical Manual of Mental Disorders, 4th edition (DSM-IV) [14], the National Institute of Neurological Disorders and Stroke, and the Association Internationale pour la Recherche et l’Enseignement en Neurosciences (NINDS-AIREN) [15].

Participants’ inclusion criteria are described as follows:

1. Dementia
  - Global cognitive impairment and defects in at least one other cognitive domain such as executive function/attention, information processing speed, visual spatial ability, language and memory, which can be confirmed by clinical and neuropsychological assessment, and
  - Cognitive impairment severe enough to interfere daily living and functioning
2. Cerebral vascular diseases, such as clear neuroimaging (MRI or CT) evidence of ischemic stroke, including infarct in the main blood vessels, single strategic infarct (e.g., thalamus, angular gyrus, and basal forebrain), multiple lacunar infarcts, and/or extensive white matter damage surrounding ventricles (≥25 % of all white matter area). Focal neurological signs that can be explained by stroke are also necessary, such as hemiplegia, anesthesia, hemianopia, dysarthria, facial paralysis, and positive reflex of Babinski’s sign
3. Correlation between dementia and cerebral vascular diseases
  - The dementia occurs within 3 months following a stroke, or
  - Evidence of sudden onset, stepwise progression, focal cortical deficits on neuropsychological assessment, and
  - Hachinski Ischemic Scale (HIS) score >4 [16] and 6-month VaD duration before inclusion
4. Severity of dementia assessed as mild to moderate as defined by a score of 11 to 26 on the MMSE [13]
5. Patients must have adequate vision and hearing to participate in study assessments

Patients who confirm any of the following excluding criteria conditions will not be enrolled in the study: subjects with AD and any other secondary types of dementia (e.g., post-traumatic dementia, dementia associated with Parkinson’s disease, or cerebral tumor); depressive pseudodementia and other mental disorders; a history of epilepsy; patients suffering from psychotic episodes; psychomotor excitation; MRI scans not showing vascular lesions in the brain; a history of drug or alcohol abuse in the past 6 months; acute or uncontrolled chronic illnesses; history of hypersensitivity to the treatment drugs; concomitant drugs with the potential to interfere with the study outcomes (including anticonvulsant agents, psychotropic drugs, drugs with psychiatric side effects, anticoagulants, cholinomimetic agents, short-acting benzodiazepines, or any other drugs that contain *Salvia miltiorrhiza*, *Panax notoginseng*, and/or *Borneolum syntheticum*); participation in another clinical study.

All subjects will undergo an MRI scan before they are randomized. Participants will be excluded if the MRI presents medial temporal lobe atrophy adjusted for age. Scans will be performed routinely at each site and will be considered adequate if the images are of sufficient technical quality to be read accurately for the purposes of determining study inclusion. After receiving this imaging information, investigators will consider the clinical history, examination, and laboratory evaluations to assign the designation of “probable VaD” or “possible VaD.”

### Study medication

All trial medication will be supplied by the Hutchison Whampoa Guangzhou Baiyunshan Chinese Medicine Company Limited (China). The FFDS tablets are composed of *Salvia miltiorrhiza* (DanShen in Chinese), *Panax pseudo*-*ginseng* (SanQi in Chinese), and *Borneolum syntheticum* (BingPian in Chinese). Each tablet weighs 0.3 g and contains tanshinone, salvianolic acid, panax notoginsenosides, ginsenoside Rb1, ginsenoside Rg1, and borneol. The FFDS tables were produced in a single batch (batch number: 080401, Placebo batch number: 081101) in strict compliance with standards of Good Manufacturing Practice (GMP).

During the 2-week placebo washout period, all patients will receive three placebo tablets three times per day. During the double-blind, 24-week intervention, the patients will receive either three FFDS or placebo tablets (three times per day), at least 2 hours apart from taking any routine Western medication. To preserve blinding, the placebo tablets have an identical taste and appearance to the FFDS tablets.

Concomitant use of anticonvulsants, antipsychotics, cholinomimetic drugs, anticholinergic agents, anti-Parkinson drugs, cholinesterase inhibitors, memantine, nootropic drugs, nimodipine, other cognition enhancers, or any drugs containing *Salvia miltiorrhiza* and *Panax notoginseng* will be forbidden. The investigator must record the concomitant drugs, including the name of the drug, daily dose, reason for using, and date of termination.

### Sample size calculations

Because there are few studies on the treatment of VaD, the sample size was calculated based on data from a study of AD treatment using the Alzheimer’s Disease Assessment Scale-cognitive subscale (ADAS-cog) [17]. The previous study reported that patients who received donepezil (5 mg/day) showed a mean improvement of 0.67 ± 0.51; the patients who received the placebo showed a worse score of 1.82 points. Using a one-sided test with a significance level of 0.05 and power of 90 %, the minimum number was 88 per group to detect a difference of 1.5 points on the ADAS-cog. To achieve an adequate number to detect the safety of the FFDS tablets, the number of the patients was increased to 100 per group. Considering a greater rate of discontinuation of 20 %, the sample size was increased to 120 per group to ensure adequate patients to complete the study.

The randomization is stratified according to center using SAS statistical software (version 9.13) (SAS Institute Inc., Cary, NC, USA). Balanced randomization generated by SAS statistical software is carried out in three steps (in blocks of four) by a statistician with no access to information on the patients or physicians. Patients will be randomized in a ratio of 1:1 to receive the FFDS or placebo tablets. The randomized code is generated in the randomization process and sealed in an envelope. Statisticians assign the medication group according to the randomization code. Afterwards, the sealed randomization codes and each available medication number are sent out to each center. Blinding will be broken only if a patient’s trial medication requires specific emergency treatment. Once the blinding is broken, the patient will be managed as off-trial.

### Procedures

**Fig. 1:**
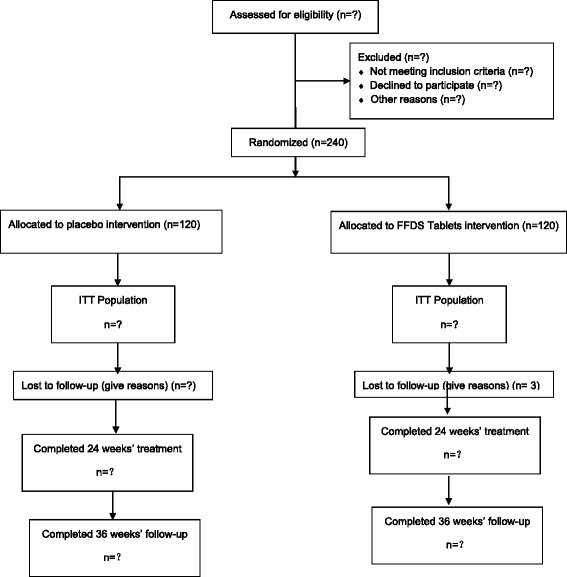
Schedule of enrollment. *ITT* intent to treat

Two methods are being used to recruit participants with VaD. The first source of subjects is from memory clinics of six public hospitals of different centers, and the second source is those who respond to advertisements published in local newspapers. Figure 1 shows the schedule of enrollment.

The neuropsychological assessments, laboratory determinations, measurements of vital signs (including temperature, blood pressure, and electrocardiogram), and neurological tests (including an examination of cranial nerves, motor coordination, muscle tonus, and power) will be evaluated before patients are enrolled. The diagnosis of probable VaD will be based on the neurological test and confirmed by the criteria of the NIND-AIREN and DSM-IV, the HIS, and the Hamilton Depression Rating Scale (HAMD) [18]. For differential diagnosis, the Activities of Daily Living (ADL) [19] and a scan of the brain are also necessary.

All patients will receive an assessment at baseline (0 week), 12 weeks, and 24 weeks, and an additional assessment will be performed 12 weeks after completing the assigned double-blind medication. The study timeline and endpoints are shown in Table 1.

**Table 1:** Study timeline and endpoints

Items	−2 weeks (run-in)	0 week (baseline)	12 weeks (mid-term follow-up)	24 weeks (endpoint)	36 weeks (follow-up)
Inclusion criteria		√			
Exclusion criteria		√			
MRI scan	√				
Informed consent	√				
General information	√				
Vital signs	√	√	√	√	√
MMSE	√	√	√	√	√
HAMD	√				
HIS	√				
ADAS-cog		√	√	√	√
CIBIS		√			
CIBIC-plus			√	√	√
ADL		√	√	√	√
Safety measure		√	√	√	
Adverse event		√	√	√	
Concomitant drugs		√	√	√	√

*CIBIS* Clinician Interview-Based Impression of Severity, *CIBIC-plus* Clinician Interview-Based Impression of Change

Any adverse events will be recorded during the duration of the trial. Patients will receive four clinical assessments.

Patients, caregivers, the study investigator, any other personnel involved in the study, and the investigating staff of the Hutchison Whampoa Guangzhou Baiyunshan Chinese Medicine Company Limited will remain blinded until all patients complete the study and all data are collected.

### Efficacy measurements

The primary efficacy measurements are the ADAS-cog [20] and the Clinician Interview-Based Impression of Change (CIBIC-plus) [21]. The ADAS-cog contains 12 items dealing with word recall, naming objects and fingers, commands, constructions and ideational praxis, orientation, word recognition, spoken language ability and comprehension of spoken language, word-finding difficulty, and attention. The scores of the ADAS-cog range from 0 to 75, and a higher score indicates higher impairment.

The CIBIC-plus is used to assess the global clinical status of the demented patient relative to baseline based on information from a semi-structured interview with the patient and the caregiver, and was designed specifically to evaluate the severity of cognitive dysfunctions characteristic of AD patients [21]. The score of the CIBIC-plus ranges from 1 to 7; a score of 1–3 indicates improvement, 4 means no change, and 5–7 indicates decline.

The secondary efficacy measurements include the MMSE and ADL [19]. The MMSE is used to assess the extent of cognitive dysfunction. The ADL mainly will be used to measure the basic activities and the instrumental activities of daily living. The ADL scale can be divided into the Physical Self-Maintenance Scale (PSMS) and the Instrumental Activities of Daily Living (IADL). The PSMS relates to physical activities, such as toileting, mobility, dressing, and bathing; the IADL contains eight items, such as shopping, cooking, doing laundry, handling finances, using the telephone, mode of transportation, responsibility for own medication, and housekeeping.

### Safety assessment

Studies have reported that the adverse events of FFDS tablets were as follows [22]: a total of 11 patients suffered adverse events. Five patients suffered allergic reactions, such as rash or allergic asthma; two patients suffered hypokalemia, with symptoms like bloating and fatigue; one patient also showed significant sinus bradycardia, one patient showed tuberculosis hemoptysis, one showed thrombocytopenia, and one patient suffered hematuria with back pain and fever.

Safety will be assessed at baseline (week 0), mid-study (week 12), and at the end of treatment (week 24). The safety assessment will include the following: (1) physical examination of vital signs, including breathing, heart rate, and blood pressure; (2) electrocardiography; (3) laboratory testing, including urine and blood; and (4) any adverse events that may occur, including the types of adverse events, time of occurrence, duration, treatment measures, and evaluation of the correlation between the tested drugs and the adverse event (positive, probable, possible, or not correlated); the severity of the adverse event (mild, moderate, and severe) must be evaluated. A mild adverse event is described as one that could induce mild symptoms, but the symptoms could be well tolerated, and there is no necessity to receive treatment or discontinue the study drugs. A moderate adverse event is defined as symptoms that could not be well tolerated and could affect the subject’s ADLs; a moderate adverse event means that the tested drug must be stopped. Severe adverse events include any event that is fatal, considered to be life-threatening, or requires hospitalization.

### Compliance strategy

To maximize subjects’ compliance, we will try to prevent dropouts by providing ongoing support to patients. A direct telephone line set up for this clinical trial will enable the study team to communicate personally with the patients. If a patient is lost to follow-up, we will call to ask the reason and attempt to schedule a meeting at the patient’s convenience.

### Statistical analysis

Statistical analyses will be conducted in three populations. The intent-to-treat (ITT) population will consist of all of the randomized population who take at least one dose of medication and at least one primary efficacy evaluation on treatment. The fully evaluated (FE) population will include all randomized patients who have received at least 80 % of the assigned 24 weeks of double-blind medication with a complete record of efficacy variables, with no major protocol violations. The safety set (SS) population will include all of the randomized population who receive at least one dose of the study medication, with at least one safety record post baseline.

The efficacy analysis will be conducted with the ITT and FE populations. The ADAS-cog and CIBIC-plus will be analyzed using the last observation carried forward (LOCF) method for the replacement of missing observations. The ADAS-cog and MMSE will be assessed by analysis of covariance (ANCOVA) with baseline scores as covariates and the treatment group and center as factors. The Cochran–Mantel–Haenszel test will be used for CIBIC-plus scores. The occurrence of adverse events will be analyzed using frequency calculations and descriptive statistics.

## Discussion

Currently, there is no medication approved for treating patients with VaD. This ongoing randomized, placebo-controlled, 24-week clinical trial will be the first rigorous testing of FFDS tablets for the treatment of patients with VaD. Success in this clinical trial will provide evidence for the use of FFDS tablets in the treatment of VaD.

A series of neuropathological studies have shown that the diagnostic accuracy of the NINDS-AIREN criteria is 90 %, and the sensitivity is 0.20–0.58 [23, 24]; the HIS also showed high specificity (87–95 %) and a relatively lower sensitivity for the diagnosis of VaD (43–50 %) [24, 25]. Thus, in the present ongoing study, the diagnosis of VaD is based on the criteria of the NINDS-AIREN and the HIS. Additionally, the present study uses structural neuroimaging to differentiate VaD from AD to enroll proper VaD subjects.

The United States Food and Drug Administration (FDA) and the European Medicines Agency (EMA) have issued guidelines on the criteria of efficacy with symptomatic improvement clinical trials [26–28]. Both of these guidelines emphasize three domains as efficacy endpoints: cognition endpoint, ADLs (functional endpoint), and overall clinical response as reflected by global assessment (global endpoint). Based on these guidelines, in the present study we are using each of these three endpoints as efficacy endpoints. In conclusion, the results of the present study are expected to provide evidence for the efficacy and safety of FFDS tablets for the treatment of patients with VaD.

## Trial status

The trial has been approved and registered. At the time of manuscript submission, the study has been actively enrolling subjects and, as of this writing, has a total of 212 subjects. The study is ongoing.

## Abbreviations

AD, Alzheimer's disease; ADAS-cog, Alzheimer’s Disease Assessment Scale-cognitive subscale; ADL, Activities of Daily Living; CIBIC-plus, Clinician Interview-Based Impression of Change; CONSORT, Consolidated Standards of Reporting Trials; CT, computer tomography; DSM-IV, Diagnostic and Statistical Manual of Mental Disorders, 4th edition; FE, fully evaluated population; FFDS, Fufangdanshen; HAMD, Hamilton Depression Rating Scale; HIS, Hachinski Ischemic Scale; IADL, Instrumental Activities of Daily Living; ITT, intent-to-treat population; LOCF, last observation carried forward; MMSE, Mini Mental State Examination; MRI, magnetic resonance imaging; NINDS-AIREN, National Institute of Neurological Disorders and Stroke and the Association Internationale pour la Recherche et l’Enseignement en Neurosciences; PSMS, Physical Self-Maintenance Scale; SS, safety set; VaD, vascular dementia

## Appendix

**Table 2:** Proposed elaborations of CONSORT items for randomized, controlled trials of FFDS tablets interventions

Paper section and topic	Item number	Descriptor	Reported on page number
Title and Abstract	1	How participants were allocated to interventions (e.g., “random allocation,” “randomized,” or “randomly assigned”)	1,3
		Either the title or abstract, or both, should state the herbal medicinal product’s Latin binomial, the part of the plant used, and the type of preparation	
Introduction			
Background	2	Scientific background and explanation of rationale	4,5
		Including a brief statement of reasons for the trial with reference to the specific herbal medicinal product being tested and, if applicable, whether new or traditional indications are being investigated	
Methods			
Participants	3	Eligibility criteria for participants and the settings and locations where the data were collected	5,6
		If a traditional indication is being tested, a description of how the traditional theories and concepts were maintained. For example, participant inclusion criteria should reflect the theories and concepts underlying the traditional indication	
Interventions	4	Precise details of the interventions intended for each group and how and when they were actually administered	
	4A: Herbal medicinal product name	1. The Latin binomial name and the botanical authority and family name for each herbal ingredient; common name(s) should also be included	6
		2. The proprietary product name (i.e., brand name) or the extract name (e.g., EGb-761) and the name of the manufacturer of the product	
		3. Whether the product used is authorized (licensed, registered) in the country in which the study was conducted	
	4B: Characteristics of the herbal product	1. The part(s) of plant used to produce the product or extract	
		2. The type of product used (e.g., raw [fresh or dry], extract)	
		3. The type and concentration of extraction solvent used (e.g., 80 % ethanol, 100 % H_2_O, 90 % glycerin, etc.) and the ratio of herbal drug to extract (e.g., 2 to 1)	
		4. The method of authentication of raw material (i.e., how done and by whom) and the lot number of the raw material. State if a voucher specimen (i.e., retention sample) was retained and, if so, where it is kept or deposited, and the reference number	
	4C: Dosage regimen and quantitative description	1. The dosage of the product, the duration of administration, and how these were determined	6,7
		2. The content (e.g., as weight, concentration; may be given as range where appropriate) of all quantified herbal product constituents, both native and added, per dosage unit form. Added materials, such as binders, fillers, and other excipients (e.g., 17 % maltodextrin, 3 % silicon dioxide per capsule), should also be listed)	
		3. For standardized products, the quantity of active/marker constituents per dosage unit form	
	4D: Qualitative testing	1. Product’s chemical fingerprint and methods used (equipment and chemical reference standards) and who performed the chemical analysis (e.g., the name of the laboratory used). Whether a sample of the product (i.e., retention sample) was retained and if so, where it is kept or deposited	No
		2. Description of any special testing/purity testing (e.g., heavy metal or other contaminant testing) undertaken; which unwanted components were removed and how (methods)	
		3. Standardization: what to standardize (e.g., which chemical components of the product) and how (e.g., chemical processes, or biological/functional measures of activity)	
	4E: Placebo/control group	The rationale for the type of control or placebo used	6,7
	4 F: Practitioner	A description of the practitioners (e.g., training and practice experience) who are a part of the intervention	8
Objectives	5	Specific objectives and hypotheses	
Outcomes	6	Clearly defined primary and secondary outcome measures and, when applicable, any methods used to enhance the quality of measurements (e.g., multiple observations, training of assessors)	8,9
		Outcome measures should reflect the intervention and indications tested considering, where applicable, underlying theories and concepts	
Sample size	7	How sample size was determined and, when applicable, explanation of any interim analyses and stopping rules	7
Randomization Sequence allocation	8	Method used to generate the random allocation sequence, including details of any restriction (e.g., blocking, stratification)	7
Allocation concealment	9	Method used to implement the random allocation sequence (e.g., numbered containers or central telephone), clarifying whether the sequence was concealed until interventions were assigned	7
Implementation	10	Who generated the allocation sequence, who enrolled participants, and who assigned participants to their groups	7
Blinding (masking)	11	Whether or not participants, those administering the interventions, and those assessing the outcomes were blinded to group assignment. If done, how the success of blinding was evaluated	7
Statistical methods	12	Statistical methods used to compare groups for primary outcome(s); methods for additional analyses, such as subgroup analyses and adjusted analyses	10
